# A real-world cost-effectiveness study of vancomycin versus linezolid for the treatment of late-onset neonatal sepsis in the NICU in China

**DOI:** 10.1186/s12913-023-09628-9

**Published:** 2023-07-19

**Authors:** Linjun Xie, Leyun Ding, Lian Tang, Zuming Yang, Dan Wu, Wenjuan Wang, Juehui Mao, Lu Shi, Chun Liu, Lufen Duan, Jinhui Xu, Qin Zhou, Jiantong Sun, Xinyuan Ding

**Affiliations:** 1 Department of Pharmacy, the Affiliated Suzhou Hospital of Nanjing Medical University, Suzhou, China; 2 Department of Neonatology, the Affiliated Suzhou Hospital of Nanjing Medical University, Suzhou, China; 3 Children’s Hospital of Soochow University, Medical College of Soochow University, Soochow University, Suzhou, China; 4 School of Basic Medicine and Clinical Pharmacy, China Pharmaceutical University, Nanjing, China

**Keywords:** Neonatal sepsis, Vancomycin, Linezolid, Real-world, Cost-effectiveness analysis

## Abstract

**Background and objective:**

Currently, the detection rates of methicillin-resistant S. aureus (MRSA) and methicillin-resistant coagulase-negative staphylococci (MRCoNS) in the blood cultures of neonates with sepsis exceed the national average drug resistance level, and vancomycin and linezolid are the primary antibacterial drugs used for these resistant bacteria according to the results of etiological examinations. However, a comprehensive evaluation of their costs and benefits in late-onset neonatal sepsis in a neonatal intensive care unit (NICU) has not been conducted. This study aimed to compare the cost and effectiveness of vancomycin and linezolid in treating neonatal sepsis in the NICU.

**Methods:**

A cost-effectiveness analysis of real-world data was carried out by retrospective study in our hospital, and the cost and effectiveness of vancomycin and linezolid were compared by establishing a decision tree model. The drug doses in the model were 0.6 g for linezolid and 0.5 g for vancomycin. The cost break down included cost of medical ward, NICU stay, intravenous infusion of vancomycin or linezolid, all monitoring tests, culture tests and drugs. The unit costs were sourced from hospital information systems. The effectiveness rates were obtained by cumulative probability analysis. One-way sensitivity analysis was used to analyze uncertain influencing factors.

**Results:**

The effectiveness rates of vancomycin and linezolid in treating neonatal sepsis in the NICU were 89.74% and 90.14%, respectively, with no significant difference. The average cost in the vancomycin group was ¥12261.43, and the average cost in the linezolid group was ¥17227.96. The incremental cost effectiveness was ¥12416.33 cost per additional neonate with treatment success in the linezolid group compared to vancomycin group at discharge. Factors that had the greatest influence on the sensitivity of the incremental cost-effectiveness ratio were the price of linezolid and the effectiveness rates.

**Conclusions:**

The cost for treatment success of one neonate in linezolid group was ¥5449.17 more than that in vancomycin group, indicating that vancomycin was more cost-effective. Therefore, these results can provide a reference for a cost effectiveness treatment scheme for neonatal sepsis in the NICU.

**Supplementary Information:**

The online version contains supplementary material available at 10.1186/s12913-023-09628-9.

## Background

Neonatal sepsis is a systemic condition of bacterial, viral, or fungal origin and leads to serious morbidity and mortality [1]. Between 1996 and 2015, the cause-specific mortality of neonatal sepsis in China decreased from 0.4 to 0.1 per 1000 neonates, which was substantially lower than the global estimate of 2.8 per 1000 neonates [2]. However, there is a serious lack of data on the costs/health care resources associated with neonatal sepsis. Gram-positive bacteria are important pathogenic bacteria in neonatal sepsis [3]. Coagulase-negative staphylococci rank first and account for more than 70% of methicillin-resistant bacteria [4]. In contrast to the drug resistance data reported by the China Antimicrobial Resistance Surveillance System (CARSS) in 2017, the detection rates of methicillin-resistant S. aureus (MRSA) and methicillin-resistant coagulase-negative staphylococci (MRCoNS) in the blood cultures of neonates with sepsis exceeded the national average drug resistance level.

Vancomycin is a type of glycopeptide antibiotic that inhibits bacterial cell wall synthesis and is used for MRSA infection. Linezolid is a type of oxazolidinone antibiotic that prevents the formation of the 70S initiation complex, which is essential for the bacterial translation process, and is an alternative antibiotic for MRSA. Both drugs have bacteriostatic and bactericidal activity against many kinds of gram-positive bacteria, including methicillin-resistant Staphylococcus aureus and methicillin-resistant coagulase-negative staphylococci [5]. Vancomycin and linezolid are important antimicrobials for severe late-onset neonatal sepsis, although efforts should be made to control the overuse of antimicrobials [6]. Treatment of neonatal sepsis with vancomycin and linezolid in the NICU has been poorly reported and requires real-world research in China. We reported that the effectiveness rates of vancomycin and linezolid in the treatment of neonatal sepsis in NICUs in China was equivalent [7]. However, the drug costs and treatment costs of vancomycin and linezolid, especially the costs involved in monitoring the blood concentration of vancomycin [8], have not yet been compared in China. Thus, the more cost effectiveness scheme needs to be further studied.

Our study conducted CEA and one-way sensitivity analysis of vancomycin versus linezolid in the treatment of neonatal sepsis in the NICU based on a real-world study. We found that the effectiveness rates of vancomycin and linezolid in the treatment of neonatal sepsis in the NICU was equivalent. The objective of this study was to evaluate the cost-effectiveness of 0.5 g vancomycin versus 0.6 g linezolid for the treatment of late-onset neonatal sepsis.

## Methods

### Model structure

A decision tree model was established based on a real-world study. The use of vancomycin for neonatal sepsis was compared with the use of linezolid. This analysis was conducted from the perspective of the China’s health care system. The time horizon covered the length of hospital stay [9].

### Interventions and comparators

The drug doses in the model were linezolid 10 mg/kg every 12 h intravenously and vancomycin 10 mg/kg every 12 h intravenously.

### Data collection and patient selection

This study was conducted at the Affiliated Suzhou Hospital of Nanjing Medical University. The neonates we recruited were aged 0 to 28 days. Patient data were collected prospectively from June 2014 through June 2020. Neonatal sepsis was defined as the growth of gram-positive bacteria in one or more blood cultures from a patient with fever (body temperature ≥ 38 °C). If the patient had multiple episodes of gram-positive bacteremia during the study period, only the first episode was included. Patients who had gram-positive bacteremia and were prescribed parenteral vancomycin or linezolid were included. Only late-onset neonatal sepsis cases were included in this study. A total of 220 patients were diagnosed with late-onset neonatal sepsis in the NICU, whose blood cultures showed gram-positive cocci. They had clinical symptoms such as abdominal distension, gastric retention, high fever, apnea, respiratory distress, and tachycardia. Patients who received medication for less than 7 days were completely excluded. Each patient’s individual clinical data were collected, such as sex, postnatal age of medication initiation, weight on medication, gestational age, Apgar score, duration of therapy, disease type, and pathogenic bacteria. The test indices before medication initiated were also collected, such as white blood cell, neutrophil, C-reactive protein, procalcitonin, hemoglobin, platelet, total bilirubin, albumin, alanine aminotransferase, and creatinine levels. All protocols were approved by the ethics committee of the Affiliated Suzhou Hospital of Nanjing Medical University in 2012, and ethical approval No. (L20129901).

### Model inputs

The Guiding Principles for Clinical Research of Antibiotics established by China Health Bureau was used to define treatment outcome, which was the highest level of agreement in China. Two researchers independently judged the treatment outcome, and the third researcher discussed or resolve the differences. The experts in the field of neonatal sepsis made the final judgment on the treatment results to ensure their accuracy [7, 10]. The curative effects of antibacterial drugs were classified into four grades: cure, improved, ineffective and progressive. “Cure” was defined as the resolution of clinical symptoms and two negative blood cultures. Clinical symptoms included abdominal distension, gastric retention, high fever, apnea, respiratory distress, and tachycardia. “Improved” was defined as clinical symptom improvement and patient discharge from the hospital. “Ineffective” was defined as no remission of symptoms and a prolonged treatment time. “Progressive” was defined as an increased number of clinical symptoms and even death. Cured and improved patients were defined as a “success”, and ineffective and progressive patients were defined as a “failure”. The effective rate was calculated as follows: effectiveness rates = successful cases/total cases × 100%. Our study adopted the perspective of the medical and health system, and the cost break down included cost of medical ward, NICU stay, intravenous infusion of vancomycin or linezolid, all monitoring tests, culture tests and drugs [11]. We identified these frequencies and unit costs from hospital information system (HIS), which is adopted by most hospitals in China. The Clinical Information System contained in HIS was used to collect and process clinical medical information of patients. We then searched the keywords for diagnosis test and drug, and obtained the frequencies and resource categories identified in the data. The actual dose for neonate was lower than the minimum drug unit regardless of vancomycin and linezolid, and the drug cost was calculated according to the price of the unit drug. Hence, we here collected the price of unit drug to calculate the drug cost.

### Cost-effectiveness analysis

After establishing the decision tree model, the patients were split into two groups: success and failure. For each treatment arm, two clinical outcomes were modeled, success and failure, and probabilities for the different parameters were determined from our retrospective hospital data [12]. The average cost was calculated by the accumulated probability and total cost [13]. The cost year was 2020, and the cost currency was ¥. The time horizon of average clinical benefit and average cost was 14 to 90 days. We performed a cost‐effectiveness analysis by calculating the ICER. In general, a higher value of the ICER indicates a less cost‐effective treatment. The ICER was measured in terms of cost spent on linezolid group and vancomycin group relative to the effectiveness rate for the treatment of late-onset neonatal sepsis:

$$ICER=\frac{Costs~of~linezolid~group~per~patient-Costs~of~vancomycin~group~per~patient}{Effectiveness~rate~of~linezolid~group-Effectiveness~rate~of~vancomycin~group}$$

### Sensitivity analysis

One-way sensitivity analysis was conducted to assess the robustness of the model results and find the key cost drivers. We conducted a series of one-way sensitivity analyses to evaluate the sensitivity of the model results to changes in the value of individual model parameters that were expected to have some impact on the overall results. The factors included the unit cost of vancomycin, unit cost of linezolid, effectiveness rates of vancomycin, and effectiveness rates of linezolid [13].

### CHEERS checklist

We analyzed the cost effectiveness of two treatments by the conventional CHEERS checklist (Table S1).

### Statistical analysis

The measurement data with a normal distribution are presented as means ± SEMs, and abnormal distributed measurement data are presented as medians (Q1, Q3). The statistical significance of continuous variables was evaluated using the Mann–Whitney U test, ANOVA and χ^2^ test with GraphPad Prism (version 6.0) and Statistical Package for the Social Sciences software (version 22.0). TreeAge Pro software was used to analyze the decision tree model. *P* < 0.05 was considered statistically significant.

## Results

### Base case analysis

**Fig. 1:**
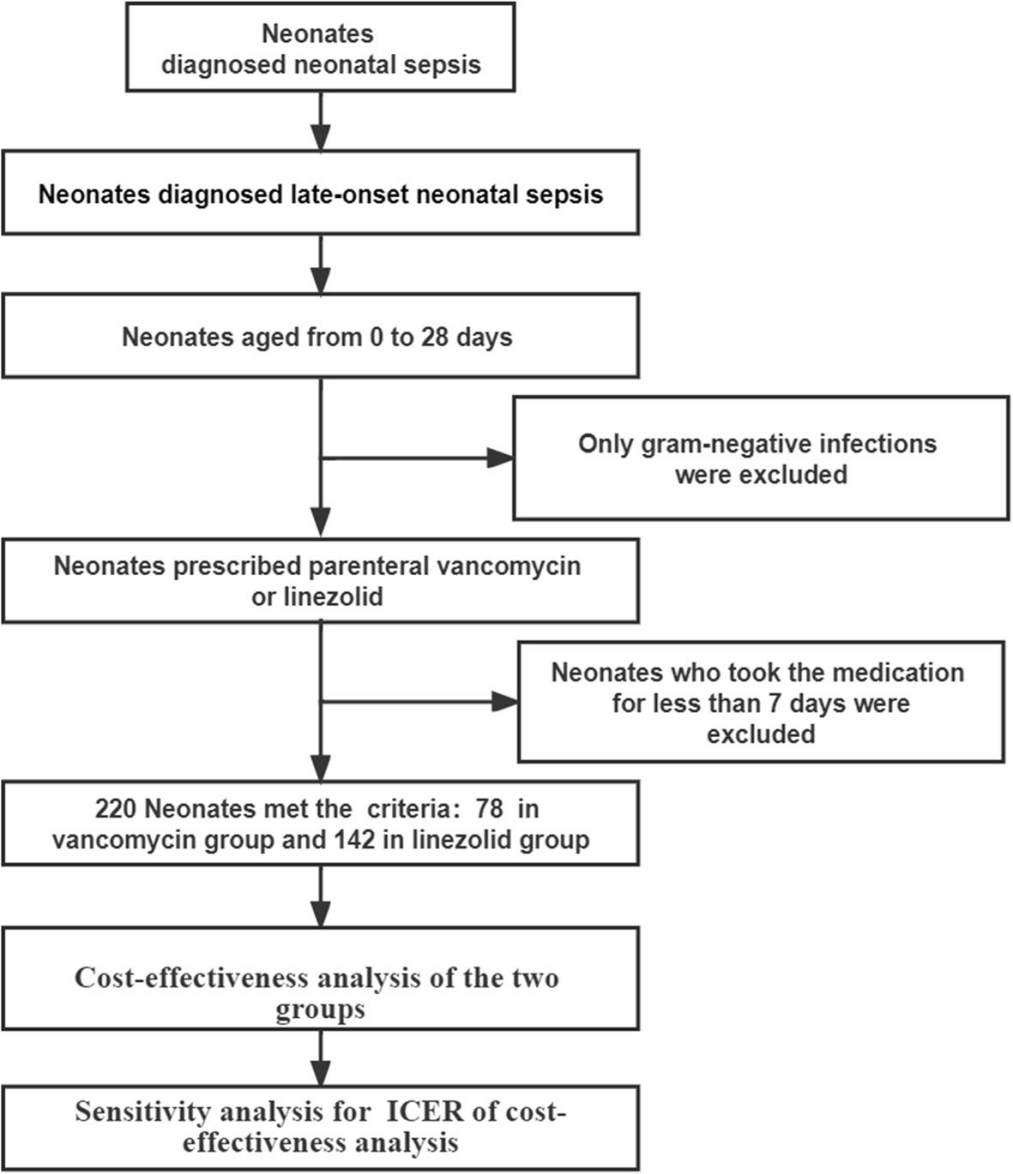
The flow diagram designed in this paper for including/excluding patients and statistical analysis of pharmaceutical economics

This study included 78 active patients in the vancomycin group and 142 in the linezolid group, all of whom were diagnosed with late-onset gram-positive bacterial sepsis (Fig. 1). There were 13 and 18 neonates in each group infected with a mixture of gram-positive bacteria. The distribution of pathogenic bacteria was dominated by coagulase-negative staphylococci, which was 50.00% in the vancomycin group and 59.86% in the linezolid group. The proportions of methicillin-resistant strains in blood cultures were 30.77% and 59.15%, respectively. There were no significant differences except for purulent meningitis (Table 1; Table S2), considering multiple interfering factors, the difference in purulent meningitis did not have an impact on the ultimate treatment effectiveness, so the treatment plans of the two groups were comparable (Table S3).

**Table 1:** Basic data comparison of the vancomycin group versus the linezolid group of patients with neonatal sepsis in our hospital

Group	Vancomycin (0.5 g)	Linezolid (0.6 g)	*P*
n	78	142	-
Sex (male/female, n)	49/29	78/64	0.234
postnatal age of medication initiation (d)	19.03 ± 1.48	15.70 ± 0.97	0.089
Weight on medication (g)	2588.19 ± 140.85	2197.97 ± 88.23	0.022
Gestational age (w)	33.70 ± 0.50	32.60 ± 0.42	0.015
1-min Apgar score [M (Q_1_, Q_3_)]	9.0 (8.0, 10.0)	9.0 (7.0, 10.0)	0.469
5-min Apgar score [M (Q_1_, Q_3_)]	9.2 (9.0, 10.0)	9.2 (8.8, 10.0)	0.520
Duration of therapy [d, M (Q_1_, Q_3_)]	14.5 (11.0, 21.0)	12.0 (10.0, 14.0)	< 0.001
Pneumonia (n)	66	127	0.370
Purulent meningitis (n)	53	6	< 0.001
Septic shock (n)	0	10	0.016
Respiratory failure (n)	40	76	0.791
Mechanical ventilation (n)	31	47	0.307

^*^P < 0.05 value was set for highly significant differences

### Costs and effectiveness

A total of 70 of 78 patients with neonatal sepsis administered 0.5 g vancomycin were successfully treated (55 cured and 15 improved), with an effective rate of 89.74%. A total of 128 of 142 patients with neonatal sepsis administered 0.6 g linezolid were successfully treated (114 cured and 14 improved), with an effective rate of 90.14%. There was no statistically significant difference between the effectiveness in the vancomycin and linezolid groups (Table S4). The per-day cost of vancomycin was ¥180.14, the per-day cost of linezolid was ¥408.47, the per-day cost of the medical ward in vancomycin group was ¥468.71, the per-day cost of the medical ward in linezolid group was ¥639.74, the intravenous infusion per-day cost of vancomycin and linezolid was ¥20.00, the cost of all monitoring tests in vancomycin group was ¥1815.70, the cost of all monitoring tests in linezolid group was ¥1293.48, and cost for culture test was ¥800.00 (Table S5). Treatment with vancomycin was estimated to be less costly than treatment with linezolid (¥12261.43 versus ¥17227.96). The date range of our study was 2014 to 2020.

### Cost-effectiveness analysis

**Fig. 2:**
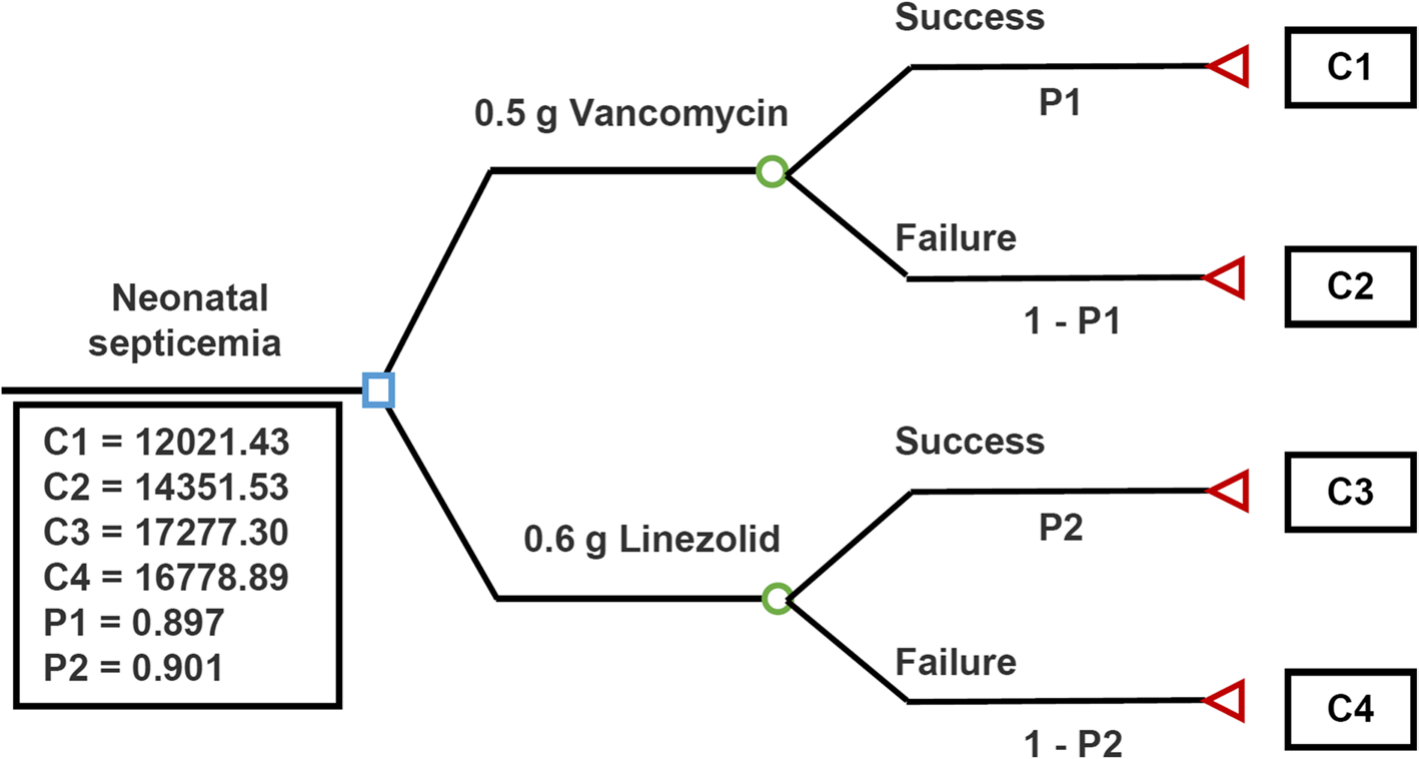
Decision tree model analysis of vancomycin group versus linezolid group of patients with neonatal sepsis in our hospital. C1: Success cost of vancomycin, C2: Failure cost of vancomycin, C3: Success cost of linezolid, C4: Failure cost of linezolid, P1: Probability of success of vancomycin, P2: Probability of success of linezolid

The average cost of the vancomycin group versus the linezolid group was shown in Fig. 2. The average cost for patients (including those who were successfully treated and those who were not) treated with 0.5 g vancomycin was lower than patients treated with 0.6 g linezolid (¥12261.43/person versus ¥17227.96/person) according to the total cost and cumulative probability analysis (Table S6; Table 2).

**Table 2:** Cost-effectiveness of the vancomycin group versus the linezolid group of patients with neonatal sepsis in our hospital

Group	Average cost (¥)	Effective rate (%)	C/E (¥/%)	Incremental cost effectiveness ratio (ICER)
Vancomycin (0.5 g)	12261.43	89.74%	13663.28	12416.33
Linezolid (0.6 g)	17227.96	90.14%	19112.45	-

The difference in average cost between the 0.5 g vancomycin and 0.6 g linezolid treatment was mostly reflected in the drug cost. The CEA showed that the effective rate in the vancomycin group was 89.74%, and the average cost was ¥12261.43. The effective rate in the linezolid group was 90.14%, and the average cost was ¥17227.96. It was ¥12416.33 cost per additional neonate with treatment success in the linezolid group compared to vancomycin group at discharge. (Table 2).

### Sensitivity analysis

**Fig. 3:**
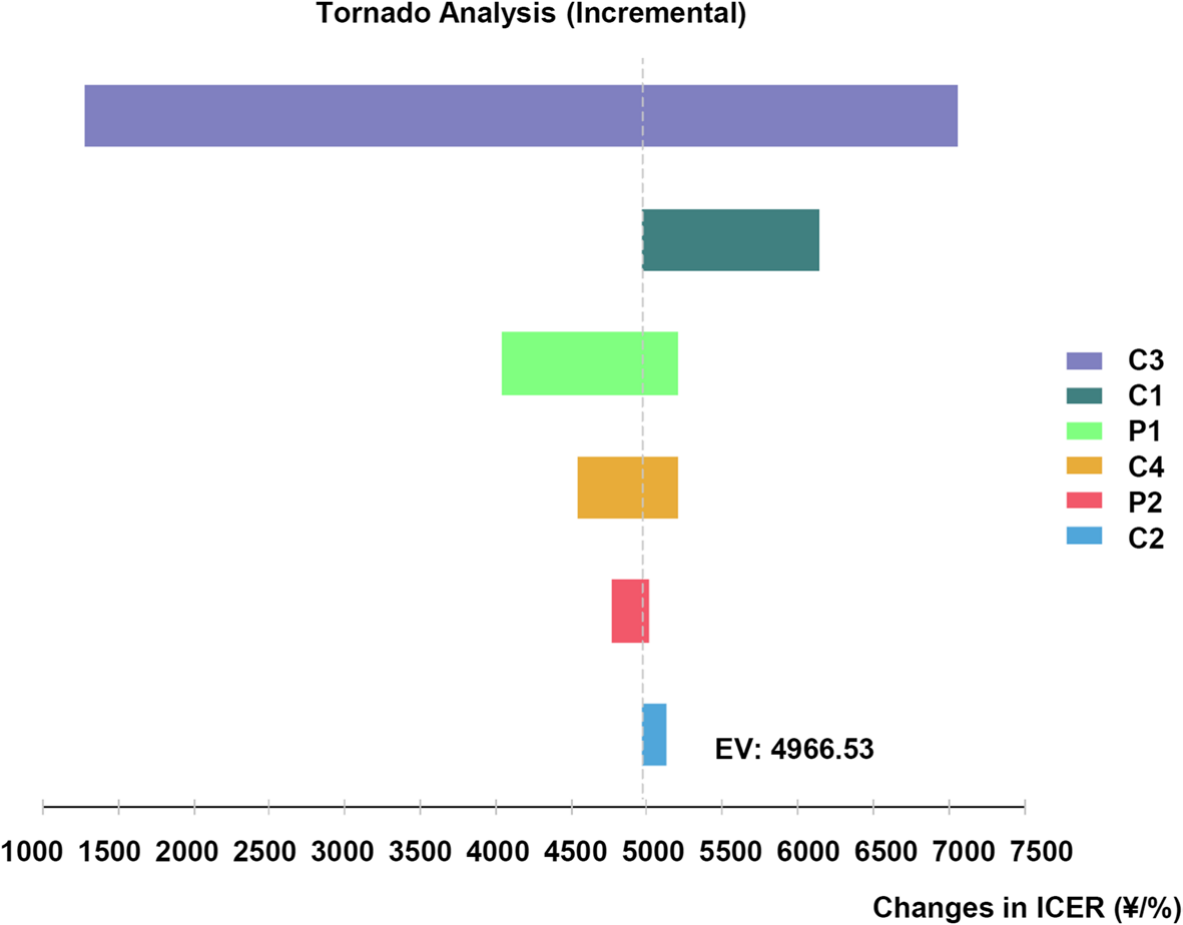
Tornado diagram of different influencing factors on the results of sensitivity of incremental cost-effectiveness ratio (ICER). Tornado diagram represented the ICER gained in the one-way sensitivity analysis for linezolid versus vancomycin. The width of the bars represented the range of the results when the variables were changed. The drug prices were derived from publicly drug procurement platform, and the effectiveness rates were cited from references. ICER = incremental cost-effectiveness ratio. C1: Success cost of vancomycin, C2: Failure cost of vancomycin, C3: Success cost of linezolid, C4: Failure cost of linezolid, P1: Success of vancomycin, probability, P2: Success of linezolid, probability

The tornado diagram depicts the effect of each input across the range of fluctuations analyzed by one-way sensitivity analysis (Fig. 3). As shown in the diagram, three uncertain factors that had the greatest influence on the sensitivity of the ICER were the price of linezolid (¥71.00 ~ ¥351.21), the price of vancomycin (¥57.69 ~ ¥103.99), and the effectiveness rates of patients treated with 0.6 g linezolid (83.67% ~ 94.65%).

Then, we performed a sensitivity analysis for these three factors. The drug cost of linezolid was analyzed in subgroups according to its two specifications. The sensitivity analysis showed that even if 0.6 g linezolid had the lowest price, its cost-effectiveness ratio was still higher than that of 0.5 g vancomycin, which had the highest price, indicating that 0.5 g vancomycin had more cost effectiveness advantages than 0.6 g linezolid (Table 3).

**Table 3:** Sensitivity analysis of vancomycin versus linezolid for patients with neonatal sepsis

Group	Vancomycin (0.5 g)		Linezolid (0.6 g)	
	Lowest	Highest	Lowest	Highest
Cost of drug daily (¥)	57.69	103.99	197.10	351.21
Average cost (¥/person)	10925.42	12261.43	16013.10	19559.67
Effective rate (%)	89.74	89.74	90.14	90.14
Cost-effectiveness ratio (¥/%)	12179.96	13663.28	17772.58	21708.85

**Fig. 4:**
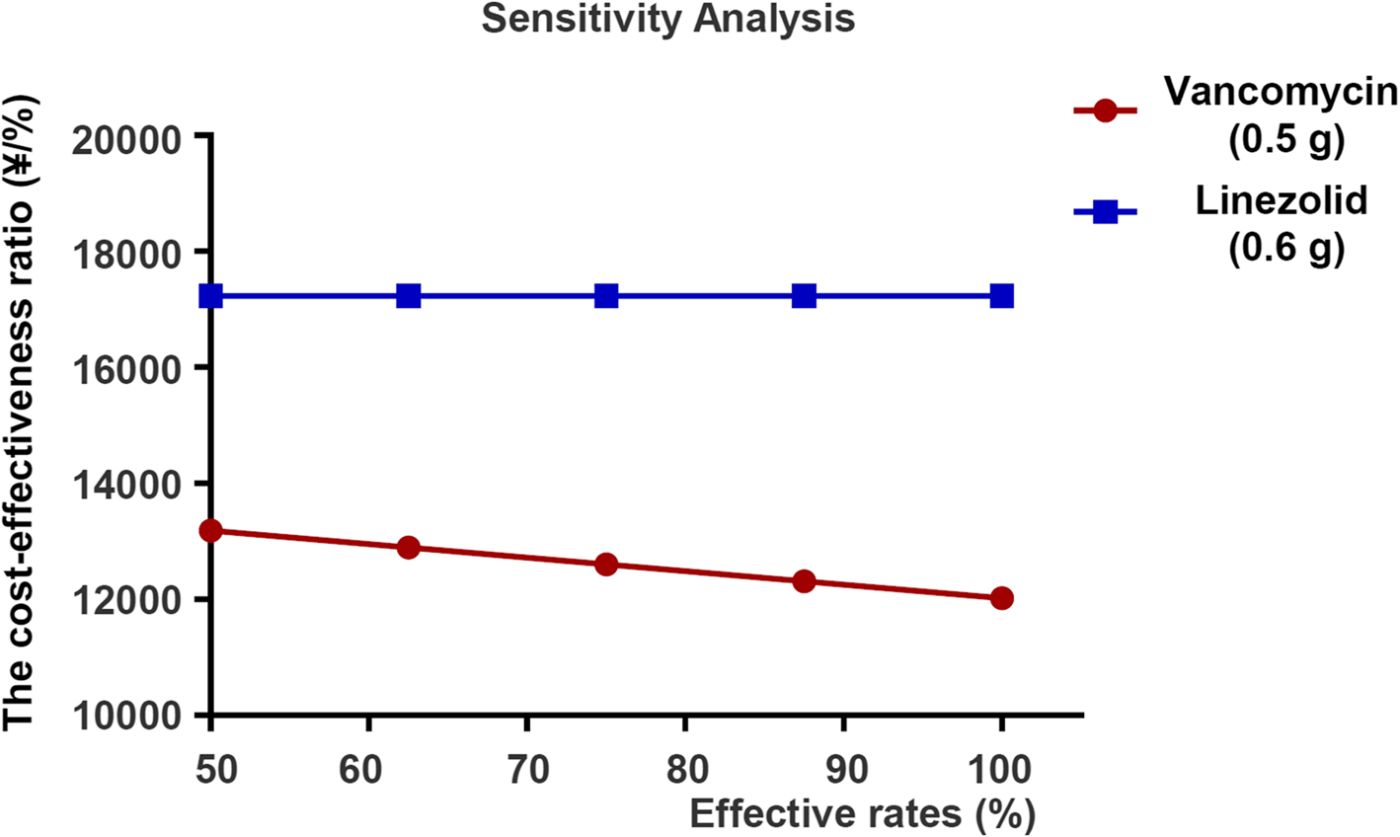
Sensitivity analysis of the effectiveness rates of treatments for patients with neonatal sepsis treated with vancomycin. According to one-way sensitivity analysis, the cost-effectiveness ratio of 0.6 g linezolid was kept as invariable and the effectiveness rates of 0.5 g vancomycin was kept as variable

Considering that the price of drugs has little effect on the effectiveness of treatment, the sensitivity analysis of effectiveness rates was conducted on patients treated with 0.5 g vancomycin versus 0.6 g linezolid. As the effectiveness rates of linezolid changed, the cost-effectiveness ratio of linezolid was always higher than that of vancomycin when the cost-effectiveness ratio of vancomycin was fixed (Fig. 4).

## Discussion

Vancomycin and linezolid are both important drugs for neonatal sepsis in the NICU. At present, there is no economic evaluation of the use of both drugs for neonatal sepsis in neonatal intensive care units. Thus, further economic research is needed to find a superior economic treatment scheme for patients with neonatal sepsis in the NICU. Here, we found that vancomycin and linezolid indeed had the same effect in treating neonatal sepsis in the NICU. With changes in linezolid specifications, the superior treatment scheme of vancomycin and linezolid in treating neonatal sepsis in the NICU changed.

Real-world studies can exhibit the cost effectiveness differences between different treatment schemes in China more directly and truly [14]. Real-world studies of vancomycin and linezolid for neonatal sepsis in NICUs in China have not been reported recently. Our study was a retrospective analysis of 0.5 g vancomycin and 0.6 g linezolid for the treatment of 220 patients with neonatal sepsis in the NICU in our hospital. Although the inclusion criteria were established, there was a significant difference between the two groups at baseline due to sex. The reason for the baseline difference might be the sample sizes of the vancomycin and linezolid groups, which were 78 and 142, respectively. Because the sample sizes were small, there might have been a serious imbalance in the sex of newborns. However, sex is not a factor that is considered in the selection of clinical treatment for neonatal sepsis [15], and it is also not a high-risk factor for neonatal sepsis [16]. Therefore, the analysis results of this real-world study were not affected by the sex of the newborns. Moreover, there was no significant difference between other clinical features, indicating that the pharmacoeconomic comparison of these two strategies in our hospital was feasible.

It was reported that CEA was employed to research the use of linezolid versus vancomycin in the empiric treatment of nosocomial pneumonia [17] and to survey the use of fidaxomicin versus vancomycin in patients with clostridium difficile infection [18]. All these studies employed CEA to evaluate the economics of the treatment of real-world diseases, which proved the feasibility of CEA. However, the CEA of vancomycin versus linezolid in the treatment of neonatal sepsis based on real-world research has not been studied before.

The difference in average total medical costs in the vancomycin and linezolid groups was mostly reflected in the drug costs. The costs of treatment programs were fixed in our hospital. However, the drug costs for patients with neonatal sepsis were different. In China, if the drug dose used is less than the minimum unit packaging dose, the unit drug price determines the drug cost. The drug dose for neonates was much less than the minimum unit packaging dose. The dose of linezolid for neonatal sepsis was much less than 0.2 g, which would not impact effectiveness and only impact the cost. In the sensitivity analysis, we found final CEA conclusion of using various specifications was totally different. Hence, it was very reasonable that the sensitivity analysis of linezolid should be conducted for different specifications. In our study, 0.5 g vancomycin was more cost-effectiveness than 0.6 g linezolid in our hospital and was supplemented by a sensitivity analysis.

Our retrospective review was in accordance with international norms, and we also consulted many relevant references. However, there were some limitations. First, as the samples in this study were collected in our hospital and the sample size was limited, the results need to be verified in multiple centers [19]. Second, it was reported that patients with neonatal sepsis often have anemia, premature birth, jaundice and other basic diseases [20, 21]. Among them, anemia was a possible adverse reaction to linezolid [22]. Since it was impossible to completely distinguish whether anemia was caused by diseases or drugs, the treatment costs for anemia were not included in the total medical costs, which interfered with the pharmacoeconomic results to a certain extent. Finally, as false-positive results may exist in the blood cultures of patients with MRCoNS infection, we tried to exclude false-positive results through clinical symptoms and bilateral double-bottle sampling to ensure the accuracy of antibiotic use.

## Conclusion

In summary, these results indicated that different drug specifications of linezolid and vancomycin had substantial economic differences in the treatment of neonatal sepsis. The effectiveness rates of vancomycin and linezolid for the treatment of neonatal sepsis in the NICU was equivalent, but treatment cost of vancomycin group was lower than linezolid group. Hence, vancomycin was more cost-effective than linezolid. Therefore, these findings will provide a clear reference for the selection of cost-effective drugs and provide clear economic options for a subset of NICU sepsis patients with unspecified bacterial infections.

## Supplementary Information

**Additional file 1:** **Table S1.** CHEERS checklist—items to include when reporting economic evaluations of health interventions.

**Additional file 2:** **Table S2.** Basic data comparison of the vancomycin group versus the linezolid group of patients with neonatal sepsis in our hospital.

**Additional file 3:** **Table S3.** Multivariate logistic regression analysis for the effect rates of vancomycin group versus linezolid group.

**Additional file 4: Table S4.** Clinical effectiveness in the vancomycin group versus linezolid group.

**Additional file 5:** **Table S5.** Base-case cost for the decision analytic model in 2020.

**Additional file 6:** **Table S6.** Total medical cost and cumulative probability analysis based on the decision tree model of the vancomycin group versus the linezolid group.

## Data Availability

The original contributions presented in this study are included in the article/Additional Material, and further inquiries can be directed to the corresponding authors.

## Abbreviations

CARSS: China antimicrobial resistance surveillance system
CEA: Cost-effectiveness analysis
ICER: Incremental cost-effectiveness ratio
MRCoNS: Methicillin-resistant coagulase-negative staphylococci
MRSA: Methicillin-resistant *S. aureus*
NICU: Neonatal intensive care unit
QALYs: Quality-adjusted life years
