# Supplementary material for: A real-world cost-effectiveness study of vancomycin versus linezolid for the treatment of late-onset neonatal sepsis in the NICU in China
Source: BMC Health Serv Res. 2023 Jul 19;23:771. doi: 10.1186/s12913-023-09628-9 (PMC10357666; doi:10.1186/s12913-023-09628-9)
Supplement: Supplementary file 1 — Additional file 1: Table S1. CHEERS checklist—items to include when reporting economic evaluations of health interventions. [file 12913_2023_9628_MOESM1_ESM.docx]

**Table S1.** CHEERS checklist—items to include when reporting economic evaluations of health interventions.

| Section/item | Item no. | Recommendation | Reported on page no./line no. |
| --- | --- | --- | --- |
| *Title and abstract* | | | |
| Title | 1 | Cost-effectiveness of vancomycin and linezolid in the treatment of neonatal sepsis. | Page 1/line 1 |
| Abstract | 2 | To investigate the cost-effectiveness of vancomycin and linezolid in the treatment of neonatal sepsis. The perspective of the study was medical care system. A retrospective study was conducted to collect data on costs and outcomes of patients from 2014 to 2020 in our hospital. The cost for treatment success of one neonate in linezolid group was ¥5449.17 more than that in vancomycin group, indicating that vancomycin was more cost-effective. | Pages 2-3/  lines 17-46 |
| *Introduction* | | | |
| Background and objectives | 3 | There are many kinds of pathogenic bacteria causing neonatal sepsis in China, which gram-positive bacteria are common. There are no drug-resistant strains on vancomycin and linezolid in China. Vancomycin and linezolid are used in the treatment of gram-positive bacterial neonatal sepsis increasingly. The cost-effectiveness between the two strategies in neonatal sepsis is still unknown. Our study helps patients make a choice on the strategy. | Pages 4-5/  lines 50-83 |
| *Methods* | | | |
| Target population and subgroups | 4 | We collected the patients of neonatal sepsis in the Affiliated Suzhou Hospital of Nanjing Medical University from June 2014 through June 2020, who used vancomycin or linezolid in the treatment. 78 patients used vancomycin, and 142 patients used linezolid. | Pages 5-6/  lines 93-113 |
| Setting and location | 5 | The study was conducted in a hospital to investigate the advantage strategy of neonatal sepsis. | Page 5/  lines 85-89 |
| Study perspective | 6 | The perspective of the study was medical care system. | Page 5/line 88 |
| Comparators | 7 | The drug doses in the model were linezolid 10 mg/kg every 12 hours intravenously and vancomycin 10 mg/kg every 12 hours intravenously. The cost-effectiveness between the two strategies in neonatal sepsis is still unknown. | Page 5/  lines 90-92 |
| Time horizon | 8 | The time horizon was almost 14 to 90 days. | Page 8/  lines 147-148 |
| Discount rate | 9 | Due to the short period of treatment, we did not consider the discount rate in this study. | NA |
| Choice of health outcomes | 10 | According to the “Guiding Principles for Clinical Research of Antibiotics”, the effects of antimicrobial drugs are classified into four grades: cure, improved, ineffective and progressive. Cure and improved are considered as “success”, and ineffective and progressive are considered as “failure”. Effectiveness rates = successful cases/total cases × 100%. | Page 7/  lines 115-129 |
| Measurement of effectiveness | 11a | Single study-based estimates. Our study based on real word and all the data can [directly](javascript:;) [manifest](javascript:;) how different drugs affect the cost-effectiveness in treatment of neonatal sepsis. | Page 7/  lines 115-119 |
|  | 11b | Synthesis-based estimates. | NA |
| Measurement and valuation of the preference based outcomes | 12 | Not applicable | NA |
| Estimating resources and costs | 13a | Single study-based economic evaluation. Our study used decision tree analysis model. The patients were split into two groups: success and failure. For each treatment arm, two clinical outcomes were modelled, success and failure, and probabilities for the different parameters were determined from our retrospective hospital data. The incremental cost-effectiveness ratio (ICER) was measured in terms of cost spent on linezolid group and vancomycin group relative to the effectiveness rate for the treatment of late-onset neonatal sepsis. | Pages 7-8/  lines 130-140 |
|  | 13b | Model-based economic evaluation. | NA |
| Currency, price date, and conversion | 14 | The cost year was 2020, and the cost currency was ¥. | Page 8/  lines 146-147 |
| Choice of model | 15 | The decision tree analysis model can easily describe the process of treatment on neonatal sepsis. | Figure 1 |
| Assumptions | 16 | After treatment, patients were split into two groups: success and failure. For each treatment arm, two clinical outcomes were modelled, success and failure, and probabilities for the different parameters were determined from our retrospective hospital data. The incremental cost-effectiveness ratio (ICER) was calculated. | Figure 1 |
| Analytical methods | 17 | Cost-effectiveness analysis (CEA) is theoretically based on decision theory and the results are expressed in the cost per effectiveness. This study used cost-effectiveness analysis. | Page 8/  lines 148-154 |
| *Results* | | | |
| Study parameters | 18 | We collected the patients of neonatal sepsis in the Affiliated Suzhou Hospital of Nanjing Medical University from June 2014 through June 2020, which used vancomycin or linezolid in the treatment. | Table 1 |
| Incremental costs and outcomes | 19 | In this study, the effective rate of vancomycin was 89.74%, and the effective rate of linezolid was 90.14%, and there was no significant difference between the two strategies (*P* > 0.05). The average cost of the vancomycin (¥12261.43/person) was lower than linezolid (¥17227.96/person). Therefore, cost-effectiveness analysis showed that vancomycin was more economical than linezolid. | Page 10/  lines 184-198 |
| Characterizing uncertainty | 20a | *Single study-based economic evaluation.* The one-way sensitivity analyses revealed that the results of the model were more sensitive to the drug cost because the variable had the greatest impact on ICER, which showed that strategies would become more unfavorable as the drug cost. The sensitivity analysis showed that even if 0.6 g linezolid had the lowest price, its cost-effectiveness ratio was still higher than that of 0.5 g vancomycin, which had the highest price, indicating that 0.5 g vancomycin was more cost-effectiveness than 0.6 g linezolid. | Pages 11-12/  lines 211-227 |
|  | 20b | *Model-based economic evaluation.* | NA |
| Characterizing heterogeneity | 21 | Not applicable | NA |
| *Discussion* | | | |
| Study findings, limitations, generalizability, and current knowledge | 22 | To conclude, our study indicated that the vancomycin strategy was cost-effective compared to linezolid in common dose, in the treatment of neonatal sepsis; with minimum dose, linezolid strategy showed more cost-effective. Since it was impossible to completely distinguish whether anemia is caused by the disease itself or by drugs, the disposal cost of anemia was not included in the cost in this study, which affected the analysis and judgment of the results to a certain extent. In the sensitivity analysis, the cost-effectiveness affected by drug dose, and it is due to the charging mechanism. | Pages 13-14/  lines 260-283 |
| *Other* | | | |
| Source of funding | 23 | Not applicable | NA |
| Conflicts of interest | 24 | Not applicable | NA |
