# Supplementary material for: A real-world cost-effectiveness study of vancomycin versus linezolid for the treatment of late-onset neonatal sepsis in the NICU in China
Source: BMC Health Serv Res. 2023 Jul 19;23:771. doi: 10.1186/s12913-023-09628-9 (PMC10357666; doi:10.1186/s12913-023-09628-9)
Supplement: Supplementary file 2 — Additional file 2: Table S2. Basic data comparison of the vancomycin group versus the linezolid group of patients with neonatal sepsis in our hospital. [file 12913_2023_9628_MOESM2_ESM.docx]

**Table S2.** Basic data comparison of the vancomycin group versus the linezolid group of patients with neonatal sepsis in our hospital.

| Group | Vancomycin (0.5 g) | Linezolid (0.6 g) | *P* |
| --- | --- | --- | --- |
| n | 78 | 143 | - |
| Pathogenic bacteria (n) | - | - | - |
| Coagulase-negative staphylococci (n) | 39 | 85 | 0.177 |
| *Staphylococcus aureus* (n) | 4 | 4 | 0.457 |
| Other gram-positive bacteria (n) | 18 | 35 | 0.816 |
| Methicillin-resistant *Staphylococcus aureus* (n) | 24 | 84 | <0.001 |
| Mixture of gram-negative bacteria (n) | 13 | 18 | 0.404 |
| Test index before medication [M (Q_1_, Q_2_)] | - | - | - |
| WBC count (10^9^/L) | 10.13 (5.71, 15.20) | 9.83 (5.75, 13.10) | 0.439 |
| Ns (%) | 52.00 (31.38, 61.15) | 50.50 (33.48, 69.25) | 0.338 |
| CRP (mg/L) | 11 (3, 35) | 7 (3, 18) | 0.113 |
| PCT (μg/L) | 0.57 (0.25, 4.00) | 0.70 (0.25, 9.40) | 0.556 |
| Hb (g/L) | 123.50 (107.75, 147.50) | 124.00 (110.00, 145.00) | 0.982 |
| PLTs (10^9^/L) | 231.00 (143.00, 374.25) | 202.00 (110.00, 314.00) | 0.247 |
| TBIL (μmol/L) | 65.50 (38.18, 128.10) | 54.00 (36.00, 142.95) | 0.870 |
| ALB (g/L) | 31.55 (27.58, 35.10) | 31.00 (25.95, 35.00) | 0.518 |
| ALT (U/L) | 16.50 (7.75, 23.25) | 16.00 (9.50, 22.00) | 0.892 |
| Cr (μmol/L) | 39.40 (26.40, 53.65) | 38.40 (28.30, 51.70) | 0.906 |

WBC: White blood cell; Ns: Neutrophils; CRP: C-reactive protein; PCT: Procalcitonin; Hb: Hemoglobin; PLTs: Platelets; TBIL: Total bilirubin; ALB: Albumin; ALT: Alanine aminotransferase; Cr: Creatinine.
