# Supplementary material for: A real-world cost-effectiveness study of vancomycin versus linezolid for the treatment of late-onset neonatal sepsis in the NICU in China
Source: BMC Health Serv Res. 2023 Jul 19;23:771. doi: 10.1186/s12913-023-09628-9 (PMC10357666; doi:10.1186/s12913-023-09628-9)
Supplement: Supplementary file 3 — Additional file 3: Table S3. Multivariate logistic regression analysis for the effect rates of vancomycin group versus linezolid group. [file 12913_2023_9628_MOESM3_ESM.docx]

**Table S3** Multivariate logistic regression analysis for the effect rates of vancomycin group versus linezolid group.

|  | Vancomycin group (0.5 g) | Linezolid group (0.6 g) | *P* | OR | 95%Cl |
| --- | --- | --- | --- | --- | --- |
| Effect rates (%) | 89.74% | 90.14% | 0.407 | 1.988 | [0.391, 10.092] |

^*^*P* < 0.05 value was set for highly significant differences.
