# Supplementary material for: A real-world cost-effectiveness study of vancomycin versus linezolid for the treatment of late-onset neonatal sepsis in the NICU in China
Source: BMC Health Serv Res. 2023 Jul 19;23:771. doi: 10.1186/s12913-023-09628-9 (PMC10357666; doi:10.1186/s12913-023-09628-9)
Supplement: Supplementary file 4 — Additional file 4: Table S4. Clinical effectiveness in the vancomycin group versus linezolid group. [file 12913_2023_9628_MOESM4_ESM.docx]

**Table S4.** Clinical effectiveness in the vancomycin group versus linezolid group.

| Group | n | Cured | Improved | Progressive | Ineffective | Effective rate (%) |
| --- | --- | --- | --- | --- | --- | --- |
| Vancomycin (0.5 g) | 78 | 55 | 15 | 1 | 7 | 89.74 |
| Linezolid (0.6 g) | 142 | 114 | 14 | 4 | 10 | 90.14 |
| *P* |  |  |  |  |  | 0.688 |
