# Supplementary material for: A real-world cost-effectiveness study of vancomycin versus linezolid for the treatment of late-onset neonatal sepsis in the NICU in China
Source: BMC Health Serv Res. 2023 Jul 19;23:771. doi: 10.1186/s12913-023-09628-9 (PMC10357666; doi:10.1186/s12913-023-09628-9)
Supplement: Supplementary file 5 — Additional file 5: Table S5. Base-case cost for the decision analytic model in 2020. [file 12913_2023_9628_MOESM5_ESM.docx]

**Table S5.** Base-case cost for the decision analytic model in 2020.

| Description | Value (price × quantity) |
| --- | --- |
| Cost for linezolid (600 mg q12h) per day | 408.48 (204.24 × 2) |
| Cost for vancomycin (500 mg q12h) per day | 180.14 (90.07 × 2) |
| Intravenous infusion of vancomycin and linezolid per day | 20.00 |
| Cost for NICU stay per day | 80.00 |
| Cost for medical ward per day in linezolid group | 639.74 |
| Cost for medical ward per day in vancomycin group | 468.71 |
| Cost for monitoring tests in linezolid group | 1293.48 |
| Cost for monitoring tests in vancomycin group | 1815.70 |
| Cost for culture test | 800.00 |
