# Supplementary material for: A real-world cost-effectiveness study of vancomycin versus linezolid for the treatment of late-onset neonatal sepsis in the NICU in China
Source: BMC Health Serv Res. 2023 Jul 19;23:771. doi: 10.1186/s12913-023-09628-9 (PMC10357666; doi:10.1186/s12913-023-09628-9)
Supplement: Supplementary file 6 — Additional file 6: Table S6. Total medical cost and cumulative probability analysis based on the decision tree model of the vancomycin group versus the linezolid group. [file 12913_2023_9628_MOESM6_ESM.docx]

**Table S6.** Total medical cost and cumulative probability analysis based on the decision tree model of the vancomycin group versus the linezolid group.

| Route | Total cost (¥/person) | Cumulative probability | Average cost (¥/person) |
| --- | --- | --- | --- |
| 1 | 12021.43 | 0.897 | 10783.22 |
| 2 | 14351.53 | 0.103 | 1478.21 |
| 3 | 17277.30 | 0.901 | 15566.85 |
| 4 | 16778.89 | 0.099 | 1661.11 |
